# The Expression of *Shmt* Genes in Amphioxus Suggests a Role in Tissue Proliferation Rather than in Neurotransmission

**DOI:** 10.3390/cells14141071

**Published:** 2025-07-13

**Authors:** Matteo Bozzo, Emanuele Serafini, Giacomo Rosa, Virginia Bazzurro, Andrea Amaroli, Sara Ferrando, Michael Schubert, Simona Candiani

**Affiliations:** 1 Dipartimento di Scienze della Terra, dell’Ambiente e della Vita, Università di Genova, 16132 Genoa, Italy; emanuele.serafini@edu.unige.it (E.S.); giacomo.rosa@edu.unige.it (G.R.); virginia.bazzurro@unige.it (V.B.); andrea.amaroli@unige.it (A.A.); sara.ferrando@unige.it (S.F.); 2 National Biodiversity Future Center (NBFC), 90133 Palermo, Italy; 3 Laboratoire de Biologie du Développement de Villefranche-sur-Mer (LBDV), Institut de la Mer de Villefranche, Centre National de la Recherche Scientifique (CNRS), Sorbonne Université, 06230 Villefranche-sur-Mer, France; michael.schubert@imev-mer.fr

**Keywords:** glycine biosynthesis, development, evolution, serine hydroxymethyltransferase, glycinergic neurons, one-carbon metabolism

## Abstract

Serine hydroxymethyltransferases (SHMTs) are key enzymes in one-carbon metabolism, with vertebrates possessing two paralogs, cytosolic SHMT1 and mitochondrial SHMT2, implicated in nucleotide biosynthesis and glycine metabolism. In this study, we investigate the evolutionary history of animal *Shmt* genes and analyze the expression patterns of *Shmt* genes in developing amphioxus (*Branchiostoma lanceolatum*). Phylogenetic analyses indicate the presence of *Shmt1* and *Shmt2* orthologs in deuterostomes, spiralians and placozoans, which is consistent with an ancient *Shmt* gene duplication event predating bilaterian diversification. Gene expression analyses in developing amphioxus show that *Shmt2* expression is confined to the somites and absent from neural tissues. In contrast, *Shmt1* is broadly expressed across germ layers, but its transcription is restricted to tissues characterized by strong cell proliferation. Notably, *Shmt1* expression in the nervous system does not match the distribution of glycinergic neuron populations, implying a negligible role in glycine neurotransmitter synthesis. Instead, the spatial correlation of *Shmt1* expression with mitotically active domains suggests a primary function in nucleotide biosynthesis via one-carbon metabolism. These findings indicate that SHMTs predominantly support cell proliferation rather than neurotransmission in amphioxus.

## 1. Introduction

Serine hydroxymethyl transferases (SHMTs) (EC 2.1.2.1) are key enzymes in cellular metabolism that catalyze the reversible conversion of serine into glycine. This reaction requires tetrahydrofolate (THF), which is concomitantly transformed into 5,10-methylenetetrahydrofolate (5,10-methylene-THF) [1]. 5,10-Methylene-THF is a central donor of one-carbon units in biosynthetic processes, particularly in nucleotide synthesis. It also serves as a precursor to 5-methyl-THF, which contributes to methylation reactions via S-adenosylmethionine [2]. While prokaryotes have one SHMT enzyme, animals have two forms of SHMT, one cytosolic and one mitochondrial, which have overlapping but distinct roles in one-carbon metabolism [1]. While both forms are encoded by a single gene in insects [3], they are encoded by different genes in jawed vertebrates (*Shmt1*, encoding the cytosolic SHMT1, and *Shmt2*, encoding the mitochondrial SHMT2) [4]. However, it remains unclear how and when this genetic specialization arose during animal evolution.

SHMTs are implicated in and required for several biological processes, including cell proliferation and embryonic development. In the fruit fly *Drosophila melanogaster*, the complete knockout of *Shmt* arrests development during cleavage [5]. In the nematode *Caenorhabditis elegans*, the disruption of *Shmt* (known as *mel-32* in this species) alters cell cycle duration [6]. In mammals, loss of *Shmt2* function strongly affects pre-implantation development and the implantation process [7,8], while *Shmt1* mutations have been associated with neural tube defects in both mice [9] and humans [10], likely due to the involvement of SHMT1 in folate metabolism [11]. The severity of the phenotypes resulting from the complete loss of *Shmt* genes makes it challenging to perform detailed analyses of SHMT functions during development. Nevertheless, two studies in *D. melanogaster* have circumvented this shortcoming. One showed that hemizygous *Shmt* mutation affects optic lobe development through its role in one-carbon metabolism [12], while the other demonstrated that an isoform-specific *Shmt* knockout impairs sleeping behavior, due to reduced serine levels [13].

Glycine is one of the 20 conserved amino acids of the standard genetic code serving as a building block for proteins [14]. However, in bilaterian animals, this amino acid acquired an additional function, as a signaling molecule in the nervous system. In the vertebrate spinal cord, for example, glycine serves as a major neurotransmitter and is used in most inhibitory synapses [15]. Glycine also acts as a co-agonist at N-methyl-D-aspartate glutamate receptors, modulating excitatory synaptic transmission [16]. The tight regulation of glycine production and uptake by neural cells is therefore crucially important for ensuring proper nervous system activity. In vertebrates, the uptake of glycine from the synaptic space, and hence the modulation of neurotransmission, is mediated by glycine transporters (GlyTs) 1 and 2, located on the plasma membranes of neurons and astrocytes [15], while the production of glycine in the terminal boutons of glycinergic neurons is regulated by the activity of SHMT2 [17,18].

In addition to a limited number of studies on the roles of SHMTs during development, previous studies on animal SHMTs have largely been focused on their roles in cancer metabolism and on the identification of therapeutical agents targeting them [19,20]. The contribution of SHMTs to folate and amino acid homeostasis as well as their roles in neuronal glycine biosynthesis have thus received surprisingly little attention [21]. Here, we characterize the *Shmt* gene complement in the cephalochordate amphioxus (*Branchiostoma lanceolatum*), the best available proxy for the last common ancestor of chordates and an important model for inferring the evolution of vertebrate traits [22]. The central nervous system of amphioxus has a vertebrate-like organization, with distinct regions homologous to the vertebrate retina, hypothalamus–prethalamus, diencephalon–mesencephalon, and hindbrain–spinal cord [23,24,25]. At the neurochemical level, all classical vertebrate neurotransmitters, including glycine, are present in the nervous system of developing amphioxus embryos and larvae, with their distribution being restricted to discrete neuronal subpopulations [26]. This makes amphioxus an ideal model for investigating the evolution of glycinergic neurotransmission and for studying the involvement of SHMTs in glycine biosynthesis. Using a combination of phylogenetic analyses and developmental gene expression assays in amphioxus, this work provides novel insights into the functional evolution of SHMTs as key players in both the metabolic regulation of development and the control of glycinergic neurotransmission.

## 2. Materials and Methods

### 2.1. Amphioxus Spawning and Material Collection

*Branchiostoma lanceolatum* (Pallas, 1774) adults were collected in Argelès-sur-Mer (France) [27] and in the North Adriatic Sea (Italy) [28] and maintained in a seawater facility at 16–17 °C until the spawning season. Spawning was induced by a mild thermal shock: ripe animals were placed in a water bath at 23 °C for 24 h and then placed individually in plastic cups at 18–19 °C for spontaneous spawning, which normally occurs 1–3 h after dark. A detailed protocol of this procedure has previously been published [29]. Cultures of the developing embryos and larvae were obtained by in vitro fertilization, raised to the desired stage at 19 °C and subsequently fixed with 4% paraformaldehyde in MOPS-EGTA buffer for in situ hybridization [29]. Staging was performed according to reference tables [30].

### 2.2. Identification of Amphioxus Shmt Genes and Phylogenetic Analyses

*Branchiostoma lanceolatum Shmt* genes were identified by BLAT searches on genomic and transcriptomic resources [31] available on the UCSC Genome Browser (https://ucsc.crg.eu/, last accessed on 16 February 2025) using the sequences of mouse *Shmt1* and *Shmt2* genes as queries. *Branchiostoma floridae Shmt* genes were identified by tBLASTx searches on the NCBI website (https://blast.ncbi.nlm.nih.gov/Blast.cgi, last accessed on 16 June 2025). Accession numbers of the sequences are provided in Supplementary Table S1.

The sequences were aligned using ClustalW in MEGA11 [32], and positions with less than 95% site coverage were removed prior to calculating phylogenies (partial deletion option). A total of 42 amino acid sequences and 451 positions were retained in the final dataset. The alignment file used to generate the trees is included as Supplementary Table S2. Phylogenetic analyses were performed using the Neighbor Joining and Maximum Likelihood methods, as implemented in MEGA11 [32]. While the Poisson model was used for calculating the Neighbor Joining tree, for the Maximum Likelihood analysis, the most appropriate evolutionary model was inferred using the Model Selection tool implemented in MEGA11 [32], selecting models with the lowest BIC scores. The Whelan–Goldman WAG model with Gamma distribution and invariant sites (WAG+G+I) was identified as the best fit. Support for internal branches for both the Neighbor Joining and the Maximum Likelihood analysis was established by non-parametric bootstrapping in 500 replicates.

### 2.3. Gene Cloning, Probe Synthesis and In Situ Hybridization

Total RNA was extracted from embryos at different developmental stages using the RNeasy Mini Kit (Qiagen, Hilden, Germany), and cDNA was synthesized using the SuperScript III Reverse Transcriptase Kit (Thermo Fisher Scientific, Waltham, MA, USA). Partial sequences of *B. lanceolatum Shmt* genes were amplified by RT-PCR. The sequences of the primers used are reported in Table 1. Amplicons were cloned into the pCRII-TOPO vector (Life Technologies, Carlsbad, CA, USA). Plasmids were purified by using the GeneJET Plasmid Mini Preparation Kit (Thermo Fisher Scientific, Waltham, MA, USA) and linearized with an appropriate restriction enzyme to serve as a template for probe synthesis. DIG-labeled antisense riboprobes were produced from 1 µg of linearized plasmid template using the DIG RNA Labeling Kit (Roche, Basel, Switzerland) following the manufacturer’s instructions. The probe was purified using the Monarch Spin RNA Cleanup Kit (New England Biolabs, Ipswich, MA, USA) and stored at −20 °C until use.

The developmental expression patterns of amphioxus *Shmt* genes were determined by whole-mount in situ hybridization. Briefly, embryos and larvae were rehydrated, digested with 7.5 µg/mL proteinase K, treated with 0.25% and 0.5% acetic anhydride and incubated with 1 ng/µL DIG-labeled probe in hybridization buffer (50% formamide, 100 μg/mL heparin, 5X SSC, 0.1% Tween-20, 5 mM EDTA, 1 mg/mL torula yeast RNA, 1X Denhardt’s solution) at 60 °C overnight. The following day, the embryos and the larvae were washed with a series of solutions of decreasing stringency (Wash solution 1: 50% formamide, 5X SSC, 1% SDS; Wash solution 2: 50% formamide, 2X SSC, 1% SDS; Wash solution 3: 2X SSC, 0.1% Tween-20; Wash solution 4: 0.2X SSC, 0.1% Tween-20), blocked with 2 mg/mL BSA and 10% sheep serum for 2 h and incubated with 1:5000 anti-DIG AP-conjugated antibody (Roche, Basel, Switzerland) at 4 °C overnight. The staining was revealed by incubation with 2.5 μL/mL NBT (Roche, Basel, Switzerland) and 3.5 μL/mL BCIP (Roche, Basel, Switzerland) in AP buffer (100 mM sodium chloride, 50 mM magnesium chloride, 100 mM Tris-HCl pH 9.6, 0.1% Tween-20, 1 mM levamisole). A detailed protocol has previously been published [29].

The stained whole-mount embryos and larvae were mounted with glycerol and photographed using an IX71 inverted microscope (Olympus, Hamburg, Germany), equipped with a ColorViewII camera (Olympus, Hamburg, Germany). Selected embryos and larvae were counter-stained with 1% Ponceau S in 1% acetic acid, dehydrated through an ethanol series and embedded in Spurr’s resin (Merck, Darmstadt, Germany) for sectioning using an RM2145 microtome (Leica, Weitzlar, Germany) [33].

## 3. Results

### 3.1. Evolutionary History of SHMT in Metazoans

**Figure 1:**
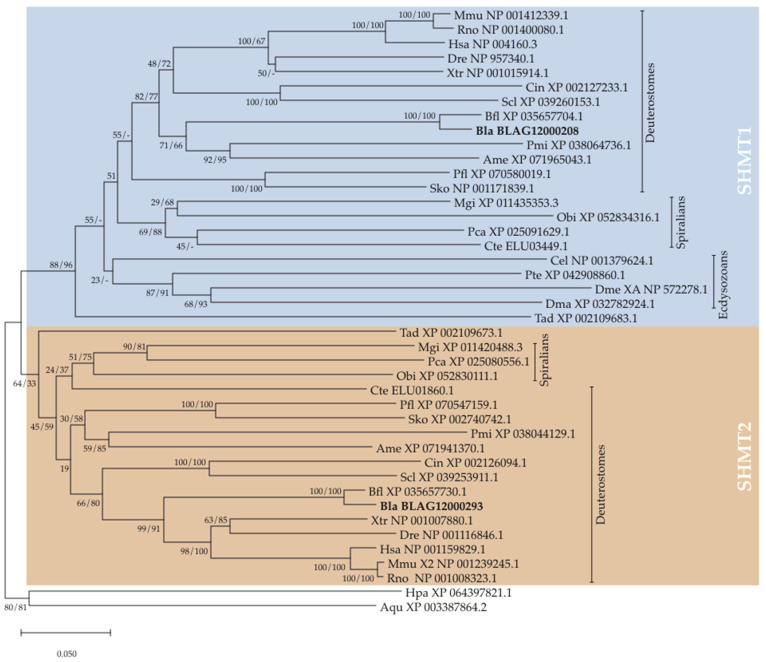
Phylogeny of metazoan SHMT proteins. Neighbor Joining and Maximum Likelihood methods were used, and the Neighbor Joining tree is shown with branch support values for the Neighbor Joining and Maximum Likelihood analyses, respectively, shown as X/Y. “-” indicates branches not recovered in the Maximum Likelihood tree. Branch lengths are expressed as amino acid substitutions per site. The tree was rooted using the sponge SHMT sequences. The *Branchiostoma lanceolatum* sequences, whose expression has been analyzed in this study, are shown in bold. List of animal species featured in the tree: Ame: *Antedon mediterranea* (sea lily, crinoid echinoderm); Aqu: *Amphimedon queenslandica* (sponge); Bfl: *Branchiostoma floridae* (Florida amphioxus, cephalochordate); Bla: *Branchiostoma lanceolatum* (European amphioxus, cephalochordate); Cel: *Caenorhabditis elegans* (roundworm, nematode); Cin: *Ciona intestinalis* (sea squirt, ascidian tunicate); Cte: *Capitella teleta* (polychaete annelid); Dma: *Daphnia magna* (water flea, crustacean arthropod); Dme: *Drosophila melanogaster* (fruit fly, insect arthropod); Dre: *Danio rerio* (zebrafish, teleost fish); Hpa: *Halichondria panicea* (sponge); Hsa: *Homo sapiens* (human, mammal); Mgi: *Magallana gigas* (Pacific oyster, bivalve mollusk); Mmu: *Mus musculus* (house mouse, mammal); Obi: *Octopus bimaculoides* (Californian two-spot octopus, cephalopod mollusk); Pca: *Pomacea caniculata* (apple snail, gastropod mollusk); Pmi: *Patiria miniata* (sea star, echinoderm); Pte: *Parasteatoda tepidariorum* (common house spider, arachnid arthropod); Rno: *Rattus norvegicus* (rat, mammal); Scl: *Styela clava* (sea squirt, ascidian tunicate); Sko: *Saccoglossus kowalevskii* (acorn worm, hemichordate); Tad: *Trichoplax adhaerens* (placozoan); Xtr: *Xenopus tropicalis* (western clawed frog, amphibian).

We identified two *Shmt* genes in each genome of the two analyzed amphioxus species, *B. floridae* and *B. lanceolatum*, and our phylogenetic analyses of SHMT sequences clearly demonstrated that these two amphioxus *Shmt* genes, respectively, encode orthologs of vertebrate SHMT1 and SHMT2 (Figure 1). We also found two *Shmt* genes in the genomes of tunicates, non-chordate deuterostomes (echinoderms and hemichordates) and spiralians (mollusks and annelids) as well as in the genome of the placozoan *Trichoplax adhaerens*. The sequences encoded by the two *Shmt* genes in these species always grouped, respectively, within the SHMT1 and the SHMT2 clade. In contrast, we only found single *Shmt* genes in arthropods and nematodes, which clustered with the SHMT1 sequences, as well as a single *Shmt* gene in each sponge genome we analyzed.

### 3.2. Expression of Shmt Genes in Amphioxus

**Figure 2:**
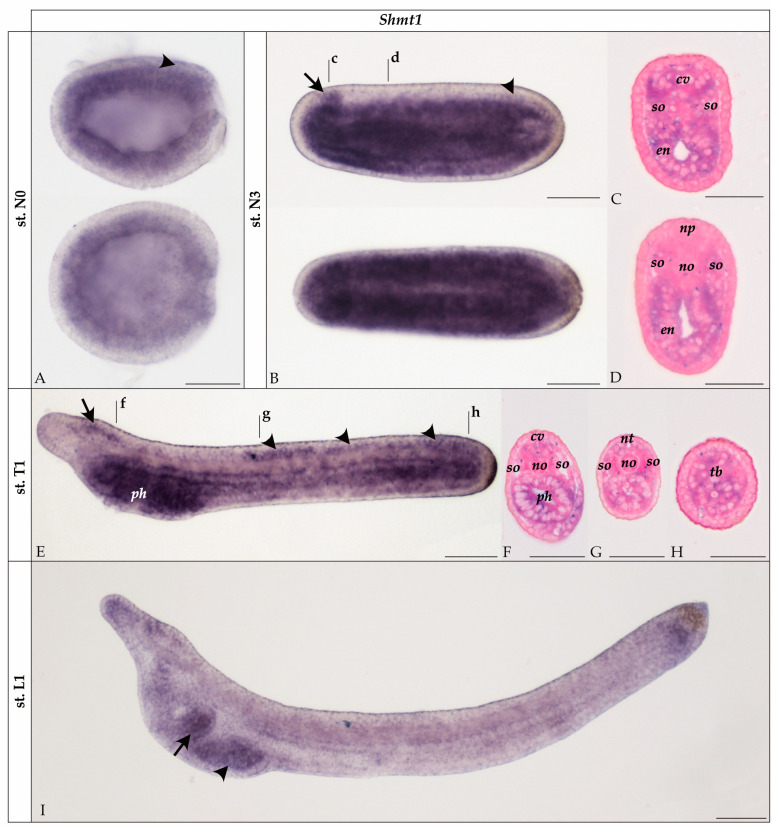
Expression of *Shmt1* in developing amphioxus (*Branchiostoma lanceolatum*). (**A**) N0 neurula in lateral (top) and dorsal (bottom) views. Arrowhead highlights expression in the posterior neural plate. (**B**) N3 neurula in lateral (top) and dorsal (bottom) views. Arrow and arrowhead, respectively, highlight expression in the cerebral vesicle and the posterior neural plate. (**C**,**D**) Transverse sections of the N3 neurula at the levels indicated by the corresponding lowercase letters in (**B**). (**E**) T1 stage embryo in lateral view. Arrow and arrowheads, respectively, highlight expression in the cerebral vesicle and the posterior rhombospinal region. (**F**–**H**) Transverse sections of the T1 embryo at the levels indicated in (**E**) by lowercase letters. (**I**) L1 larva in lateral view. Arrow and arrowhead, respectively, highlight expression in the club-shaped gland and the first pharyngeal gill slit. All whole mounts are oriented with the anterior to the left. Scale bar is 50 µm for whole mounts and 25 µm for sections. st.: stage; cv: cerebral vesicle; en: endoderm; no: notochord; np: neural plate; nt: neural tube; ph: pharynx; so: somite; tb: tail bud.

In developing *B. lanceolatum*, *Shmt1* transcripts were first detected at the N0 neurula stage, showing broad expression in the endomesoderm as well as in the posterior dorsal ectoderm (i.e., in the posterior neural plate) (Figure 2A). At the N3 stage, *Shmt1* was expressed across all germ layers, except in the general ectoderm, with strong signals in the endoderm and somites (Figure 2B–D). Prominent expression was also observed in the central nervous system, particularly in the cerebral vesicle and the posterior neural plate (Figure 2B,C).

At the T1 stage, *Shmt1* expression in the endomesoderm was still conspicuous in the pharyngeal endoderm, posterior endoderm and tail bud, but downregulated in the somites and endoderm in the center of the embryo (Figure 2E–H). At this stage, expression was also detectable in the rostral ectoderm (Figure 2E). In the central nervous system, the gene was expressed in the dorsal cerebral vesicle as well as in the rhombospinal region, most conspicuously in a territory located just posterior to the first ocellus (Figure 2E,G).

By the L1 larval stage, *Shmt1* expression was still detectable in the pharyngeal region, most notably in the club-shaped gland and the first pharyngeal gill slit, in the tail bud, in the rostral ectoderm as well as in the cerebral vesicle (dorsally and in the frontal eye complex) (Figure 2I).

**Figure 3:**
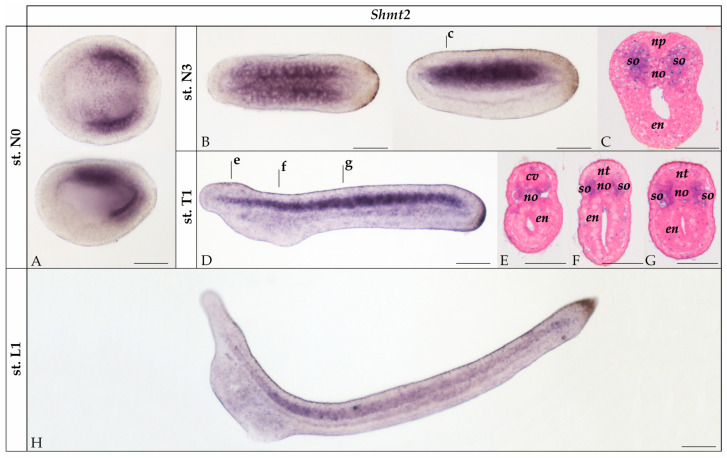
Expression of *Shmt2* in developing amphioxus (*Branchiostoma lanceolatum*). (**A**) N0 neurula whole mount in dorsal (top) and lateral (bottom) views. (**B**) N3 neurula in dorsal (left) and lateral (right) views. (**C**) Transverse section of the N3 neurula at the level indicated by the lowercase letter in (**B**). (**D**) T1 stage embryo in lateral view. (**E**–**G**) Transverse sections of the T1 embryo at the levels indicated by the corresponding lowercase letters in (**D**). (**H**) L1 larva in lateral view. All whole mounts are oriented with the anterior to the left. Scale bar is 50 µm for whole mounts and 25 µm for sections. st.: stage; cv: cerebral vesicle; en: endoderm; no: notochord; np: neural plate; nt: neural tube; so: somite.

In *B. lanceolatum* N0 neurulae, *Shmt2* expression was restricted to the lateral endomesoderm of the posterior two thirds of the embryo (Figure 3A). At the N3 stage, *Shmt2* transcripts were detected in the dorsal mesoderm, in both the notochord and the somites (Figure 3B,C). This pattern persisted at the T1 stage (Figure 3D–G). By the L1 larval stage, *Shmt2* expression was markedly reduced and restricted to the somites, with the most conspicuous signal detectable in the center of the larva (Figure 3H).

## 4. Discussion

### 4.1. Shmt Gene Duplication Took Place During Early Metazoan Evolution

In vertebrates, the SHMT1 and SHMT2 proteins are respectively encoded by the *Shmt1* and *Shmt2* genes, and the mitochondrial SHMT2 has been proposed to catalyze the synthesis of glycine, which functions as a neurotransmitter in glycinergic neurons [17,18]. We identified two *Shmt* genes in amphioxus, tunicates and ambulacrarians which, together with vertebrates, represent the totality of deuterostome lineages. Phylogenetic analyses (Figure 1) revealed that the SHMT proteins encoded by these two genes in amphioxus, tunicates and ambulacrarians are orthologs of, respectively, vertebrate SHMT1 and SHMT2. Additionally, we found distinct *Shmt1* and *Shmt2* orthologs in the genomes of mollusks and annelids as well as placozoans. This is the first report of the presence of two distinct *Shmt* genes in invertebrates, suggesting that the duplication of *Shmt* genes was not a vertebrate-specific innovation but instead reflects a more ancient condition [3]. Given that we identified only single *Shmt* genes in sponges but distinct *Shmt1* and *Shmt2* orthologs in placozoans, we hypothesize that the duplication of an ancestral *Shmt* gene into *Shmt1* and *Shmt2* has occurred very early in the metazoan lineage, likely at the base of eumetazoan animals.

Seemingly contradicting this hypothesis, previous reports suggested that the duplication of *Shmt* genes represents a lineage-specific trait of vertebrates. This conclusion was based on the presence of a single *Shmt* gene in insects [3], and, while our analyses confirmed the presence of a single *Shmt1*-like gene in insects, we further identified single *Shmt1*-like genes in other ecdysozoans, including crustaceans, arachnids and nematode worms. In the context of our expanded phylogenetic sampling recovering distinct *Shmt1* and *Shmt2* orthologs from both bilaterian and non-bilaterian animals, the most parsimonious interpretation of the absence of *Shmt2*-like genes from major ecdysozoan lineages is an ancestral loss of the *Shmt2* gene. More elaborate phylogenetic analyses incorporating SHMT sequences from additional ecdysozoan groups will be required to accurately pinpoint the timing of this gene loss event during ecdysozoan diversification.

### 4.2. SHMTs Are Not Involved in Neurotransmission in Amphioxus

In amphioxus, *Shmt1* was broadly expressed in several tissues from stage N3 to T1 (Figure 2), while *Shmt2* expression was restricted to the mesoderm and more specifically the somites (Figure 3). This tissue-restricted expression is consistent with a possible role of SHMT2 during muscle development, the main derivative of somites in amphioxus. Interestingly, *Shmt2* was not expressed in the neural tube, suggesting that it does not contribute to glycine synthesis and thus neurotransmission in amphioxus. Consequently, we shifted our focus to amphioxus *Shmt1*, despite the minor role of vertebrate SHMT1 in glycine synthesis [34]. The possibility of a functional inversion between *Shmt1* and *Shmt2* in cephalochordates and vertebrates would not be unprecedented. A similar case has been reported for the glycine transporters *GlyT1* and *GlyT2*, which, when comparing their respective developmental expression patterns in cephalochordates and vertebrates, display inverted expression domains in neurons and astroglia [35,36].

Unlike *Shmt2*, *Shmt1* was expressed in the amphioxus central nervous system as well as in other tissues (Figure 2). In zebrafish, *Shmt1* is expressed in specific regions of the developing brain, notably the retina and the hindbrain, which are rich in glycinergic neurons, as well as in the optic tectum (www.zfin.org/ZDB-GENE-040426-1558/expression, last accessed on 8 July 2025). In contrast, within the central nervous system of amphioxus, the expression of *Shmt1* did not include territories characterized by the presence of glycinergic neuron populations, which include three pairs of cells in the anterior rhombospinal region and scattered cells near the first ocellus [26]. Instead, the expression of *Shmt1* in the amphioxus central nervous system was concentrated in the dorsal cerebral vesicle and in the region posterior to the first ocellus, at the N3 and T1 stages, before becoming restricted, in L1 larvae, to the cerebral vesicle (Figure 2). Since putative glycinergic neurons in L1 larvae are located posterior to the cerebral vesicle [26], it is unlikely that SHMT1 is responsible for glycine biosynthesis in these neurons. These findings contrast with the accepted origin of glycine in vertebrate glycinergic neurons and with the observation that *Shmt* gene silencing lowers glycine levels in the glycinergic neurons of *D. melanogaster* [37]. It might be that, in amphioxus, glycine synthesis in glycinergic neurons relies on an alternative metabolic pathway, such as an inverted glycine cleavage system or GlyT-mediated glycine uptake from the extracellular space [15,38]. Further, more detailed analyses of glycine metabolism in the amphioxus nervous system will be essential to elucidate the biochemical routes responsible for the synthesis of this crucial neurotransmitter in cephalochordates.

### 4.3. SHMT1 Is Important in Proliferating Tissues During Amphioxus Development

We found that the expression patterns of *Shmt1* closely match regions of active proliferation during amphioxus development. Between the N3 and T1 stages, active proliferation occurs in the endoderm, central nervous system and posterior somites, as revealed by EdU incorporation [39]. Cell proliferation within the central nervous system is particularly conspicuous in the cerebral vesicle and the posterior rhombospinal region [39], matching *Shmt1* expression (Figure 2). This spatial correlation is coherent with a functional association of *Shmt1* expression and cell proliferation, indicating that the primary function of SHMT1 in amphioxus is more closely tied to the folate pathway and one-carbon metabolism than to the synthesis of the neurotransmitter glycine. The link between SHMT1 and mitotically active regions would thus be consistent with the known role of this enzyme in nucleotide biosynthesis. Specifically, SHMT1 catalyzes the production of 5,10-methylene-THF, a key donor of one-carbon units required for the synthesis of thymidine and purines, both essential for DNA replication and cell division [40,41].

## 5. Conclusions

Most animals possess two *Shmt* genes, one cytosolic and one mitochondrial [1,3], that, according to our phylogenetic analyses, originated by gene duplication during early metazoan evolution. Our expression analyses suggest that SHMTs do not play a primary role in glycine neurotransmitter synthesis in amphioxus. Instead, glycine production in the glycinergic neurons of amphioxus might occur via alternative SHMT-independent pathways. In developing amphioxus embryos and larvae, *Shmt1* expression is closely correlated with regions of active cell proliferation, supporting a primary role for SHMT1 in nucleotide biosynthesis by mediating one-carbon metabolism. These findings point to a potentially ancestral role for SHMTs in maintaining rapid cell division during chordate development.

## Figures and Tables

**Table 1:** Sequences of the primers used to amplify *Branchiostoma lanceolatum Shmt* genes.

Primer	Sequence
*Shmt1* forward	5′-CTACAGGCCTTGGGGTCTTG-3′
*Shmt1* reverse	5′-GGTGTTCCAAAACGCAGACC-3′
*Shmt2* forward	5′-CGTTCGTCTCCAGTTCAACC-3′
*Shmt2* reverse	5′-CTGTAAGGCATGGACTCAAAGT-3′

## Data Availability

The data supporting the findings of this study are included in the article and its Supplementary Material.

## Supplementary Materials

The following supporting information can be downloaded at https://www.mdpi.com/article/10.3390/cells14141071/s1, Table S1: Accession numbers of the sequences used for the phylogenetic analyses presented in Figure 1; Table S2: Multisequence alignment in FASTA format used for the phylogenetic analyses presented in Figure 1.

## Abbreviations

The following abbreviations are used in this manuscript:

GlyT	Glycine transporters
SHMT	Serine hydroxymethyltransferase
THF	Tetrahydrofolate
