# Supplementary material for: The Expression of Shmt Genes in Amphioxus Suggests a Role in Tissue Proliferation Rather than in Neurotransmission
Source: Cells. 2025 Jul 13;14(14):1071. doi: 10.3390/cells14141071 (PMC12293503; doi:10.3390/cells14141071)
Supplement: Supplementary file 1 [file cells-14-01071-s001.zip › cells-3739142-supplementary.pdf]

Expression of *Shmt* Genes in Amphioxus Suggests a Role in Tissue Proliferation Rather Than Neurotransmission

Matteo Bozzo [1,*], Emanuele Serafini [1], Giacomo Rosa [1,2], Virginia Bazzurro [1], Andrea Amaroli [1], Sara Ferrando [1,2], Michael Schubert [3,†] and Simona Candiani [1,*,†]

[1] Dipartimento di Scienze della Terra, dell'Ambiente e della Vita, Università di Genova, 16132 Genoa, Italy; emanuele.serafini@edu.unige.it (E.S.); giacomo.rosa@edu.unige.it (G.R.); virginia.bazzurro@unige.it (V.B.); andrea.amaroli@unige.it (A.A.); sara.ferrando@unige.it (S.F.)
[2] National Biodiversity Future Center (NBFC), 90133 Palermo, Italy
[3] Laboratoire de Biologie du Développement de Villefranche-sur-Mer (LBDV), Institut de la Mer de Villefranche, Sorbonne Université, CNRS, 06230 Villefranche-sur-Mer, France; michael.schubert@imev-mer.fr
* Correspondence: matteo.bozzo@unige.it (M.B.); candiani@unige.it (S.C.)
† These authors contributed equally to this work.

Supplementary material

Table S1. Accession numbers of the sequences used for the phylogenetic analyses presented in Figure 1.

**Species**	**Gene**	**Accession Number**	**Database**
Amphimedon queenslandica	SHMT1/2	XP_3387864.2	NCBI
Halichondria panicea	SHMT1/2	XP_64397821.1	NCBI
Antedon mediterranea	SHMT1	XP_71965043.1	NCBI
Branchiostoma floridae	SHMT1	XP_035657704.1	NCBI
Branchiostoma lanceolatum	SHMT1	BLAG12000208_1	UCSC
Caenorhabditis elegans	SHMT1	NP_001379624.1	NCBI
Capitella teleta	SHMT1	ELU03449.1	NCBI
Ciona intestinalis	SHMT1	XP_002127233.1	NCBI
Danio rerio	SHMT1	NP_957340.1	NCBI
Daphnia magna	SHMT1	XP_032782924.1	NCBI
Drosophila melanogaster	SHMT1	XA_NP_572278.1	NCBI
Homo sapiens	SHMT1	NP_004160.3	NCBI
Magallana gigas	SHMT1	XP_011435353.3	NCBI
Mus musculus	SHMT1	NP_001412339	NCBI
Octopus bimaculoides	SHMT1	XP_052834316.1	NCBI
Parasteatoda tepidariorum	SHMT1	XP_042908860.1	NCBI
Patiria miniata	SHMT1	XP_038064736.1	NCBI
Pomacea canaliculata	SHMT1	XP_25091629.1	NCBI
Ptychodera flava	SHMT1	XP_070580019.1	NCBI
Rattus norvegicus	SHMT1	NP_001400080	NCBI
Saccoglossus kowalevskii	SHMT1	XP_2740742.1	NCBI
Styela clava	SHMT1	XP_39260153.1	NCBI
Trichoplax adhaerens	SHMT1	XP_2109683.1	NCBI

Xenopus tropicalis	SHMT1	NP_001015914.1	NCBI
Antedon mediterranea	SHMT2	XP_71941370.1	NCBI
Branchiostoma floridae	SHMT2	XP_035657730.1	NCBI
Branchiostoma lanceolatum	SHMT2	BLAG12000293_1	UCSC
Capitella teleta	SHMT2	ELU01860.1	NCBI
Ciona intestinalis	SHMT2	XP_002126094.1	NCBI
Danio rerio	SHMT2	NP_001116846.1	NCBI
Homo sapiens	SHMT2	NP_001159829.1	NCBI
Magallana gigas	SHMT2	XP_011420488.3	NCBI
Mus musculus	SHMT2	X2_NP_00123924	NCBI
Octopus bimaculoides	SHMT2	XP_052830111.1	NCBI
Patiria miniata	SHMT2	XP_038044129.1	NCBI
Pomacea canaliculata	SHMT2	XP_25080556.1	NCBI
Ptychodera flava	SHMT2	XP_070547159.1	NCBI
Rattus norvegicus	SHMT2	NP_001008323.1	NCBI
Saccoglossus kowalevskii	SHMT2	XP_1171839.1	NCBI
Styela clava	SHMT2	XP_39253911.1	NCBI
Trichoplax adhaerens	SHMT2	XP_2109673.1	NCBI
Xenopus tropicalis	SHMT2	NP_001007880.1	NCBI

Table S2. Multisequence alignment in FASTA format used for the phylogenetic analyses presented in Figure 1.

```
>Hsa_SHMT1_NP_004160.3 serine hydroxymethyltransferase, cytosolic isoform 1 [Homo
sapiens]
--------------------------------------------------------MTMPVNGAHKDADLWS---------------
------------
SHDKMLAQPLKDSDVEVYNIIKKESNRQRVGLELIASENFASRAVLEALGSCLNNKYSEGYPGQRYYGGTEFIDELETLCQKRALQA
YKLDPQCWGVNVQPYSGSPANFAVYTALVEPHGRIMGLDLPDGGHLTHGFMTDKKKISATSIFFESMPYK-----------------
--------------VN-----------------------------------
PDTGYINYDQLEENARLFHPKLIIAGTSCYSRNLEYARLRKIADENGAYLMADMAHISGLVAAGVVPSPFEHCHVVTTTTHKTLRGC
RAGMIFYRKGVKSVDPKTGKEILYNLESLINSAVFPGLQGGPHNHAIAGVAVALKQAMTLEFKVYQHQVVANCRALSEALTEL-
GYKIVTGGSDNHLILVDLRSKGTDGG--RAEKVLEACSIACNKNTCPGDR-
SALRPSGLRLGTPALTSRGLLEKDFQKVAHFIHRGIELTLQIQSDTGVR---ATLKEFKERLAGD-
KYQAAVQALREEVESFASLFPLPGLPDF--
>Mmu_SHMT1_NP_001412339.1 serine hydroxymethyltransferase, cytosolic isoform 1 [Mus
musculus]
--------------------------------------------------------------MADRDATLWA---------------
------------
SHEKMLSQPLKDSDAEVYSIIKKESNRQRVGLELIASENFASRAVLEALGSCLNNKYSEGYPGQRYYGGTEFIDELEMLCQKRALQA
YHLDPQCWGVNVQPYSGSPANFAVYTALVEPHGRIMGLDLPDGGHLTHGFMTDKKKISATSIFFESMPYK-----------------
--------------VY-----------------------------------
PETGYINYDQLEENASLFHPKLIIAGTSCYSRNLDYARLRKIADDNGAYLMADMAHISGLVAAGVVPSPFEHCHVVTTTTHKTLRGC
RAGMIFYRKGVRSVDPKTGKETYYELESLINSAVFPGLQGGPHNHAIAGVAVALKQAMTTEFKIYQLQVLANCRALSDALTEL-
GYKIVTGGSDNHLILMDLRSKGTDGG--RAEKVLEACSIACNKNTCPGDK-
SALRPSGLRLGTPALTSRGLLEEDFQKVAHFIHRGIELTLQIQSHMATK---
ATLKEFKEKLAGDEKIQSAVATLREEVENFASNFSLPGLPDF--
>Rno_SHMT1_NP_001400080.1 serine hydroxymethyltransferase, cytosolic [Rattus
norvegicus]
--------------------------------------------------------------MAERDATVWA---------------
------------
SHEKMLTQPLKESDAEVYSIIKKESNRQRVGLELIASENFASRAVLEALGSCLNNKYSEGYPGQRYYGGTEFIDELETLCQKRALQA
YHLDPQCWGVNVQPYSGSPANFAVYTALVEPHGRIMGLDLPDGGHLTHGFMTDKKKISATSIFFESMPYK-----------------
--------------VY-----------------------------------
PDTGYINYDQLEENASLFHPKLIIAGTSCYSRNLDYARLRKIADDNGAYLMADMAHISGLVAAGVVPSPFEHCHVVTTTTHKTLRGC
```

```
RAGMIFYRKGVRSVDPKTGEETYYELESLINSAVFPGLQGGPHNHAIAGVAVALKQAMTTEFKIYQLQVLANCRALSDALTEL-
GYKIVTGGSDNHLILMDLRPKGTDGG--RAEKVLEACSIACNKNTCPGDK-
SALRPSGLRLGTPALTSRGLLEEDFQKIAHFIHRGIELTLQIQSHMTMR---
ATLKEFKEKLTGDEKFQSAVAALREEVENFASNFSLPGLPDF--
>Dre_SHMT1_NP_957340.1 serine hydroxymethyltransferase, cytosolic [Danio rerio]
----------------------------------------------------------MSQRNGHT-EKTWE---------------
------------
SHNKMMLEPLSTNDPEVFDIIKKEKKRQTYGLELIASENFTSRAVLEALGSCMNNKYSEGYPGQRYYGGTEHVDELERLCQDRALKV
YGLDPEKWGVNVQPYSGSRANFAVYTAIVEPHGRIMGLDLPDGGHLTHGFMTDKKKISATSIFFESMPYK-----------------
--------------VN-----------------------------------
PETGYIDYNRLEENARLFHPRLIIAGTSCYSRNLDYSRLRKIADENGAYLLADMAHISGLVAAGVVPSPFEYCDVVSTTTHKTLRGC
RAGVIFFRKGVRSVDAKTGKETMYNLESLINQAVFPGLQGGPHNHAIAGVAVALKQALTPEFKTYQLQVLANCKALASALMDK-
GYKVVTGGSDNHLILVDLRSNGTDGG--RAEKVLEACAIACNKNTCPGDK-
SALRPSGLRLGSPALTSRGLLEEHFHKVAEFIHQGIVLTLEIQKNMNPK---
ATLKEFKEELSQNEKYQLKTKEIRKEVEDFAGKFPMPGLPEL--
>Xtr_SHMT1_NP_001015914.1 serine hydroxymethyltransferase, cytosolic [Xenopus
tropicalis]
-------------------------------------------------------MADSAQINGSTHQETWE---------------
------------
SHNKMVLEPLDTNDPEVYDIIRKEKNRQRYGLELIASENFASCAVLQALGSCLNNKYSEGYPGQRYYGGTEFVDEMERLCQKRALEV
YGLDPQKWGVNVQPYSGSPANFAVYTALVEPHGRIMGLDLPDGGHLTHGFMTDKKKISATSIFFESMPYK-----------------
--------------VH-----------------------------------
PDTGYIDYDRLEENARLFHPKLIIAGVSCYSRNLDYARMRRIANENNAVLMADMAHISGLVAAGVVPSPFEHCDVVSTTTHKTLRGC
RSGMIFYRKGVRSVDPKTGKETLYNYESLINQAVFPGLQGGPHNHAIAGVAVALKQALSPEFKLYQRQVVSNCKALSSAMEEL-
GYHVVTGGSDNHLILVNLRGQKTDGG--RAEKVLEACAIACNKNTCPGDK-
SALRPSGLRLGTPALTSRGFKEDDFKKVAQFIHRGIELTLEIQNAMIPG---
ATLKDFKEKLASEDVHTPKMLALRAEVEKFAGTFPIPGLPDL--
>Bfl_SHMT1_XP_035657704.1 serine hydroxymethyltransferase, cytosolic-like
[Branchiostoma floridae]
-------------------------------------------------------MGEPMQMTNGHS--------------------
-----------
AGDWWDLTKPVGEVDPEITAIIRKEKDRQRRELEMIASENFASAACLQAMGSCLNNKYSEGYPGQRYYGGTKFVDEIEVLCQKRALS
VYGLDPEKWGVNVQPYSGSPANFAVFTAIVEPHGRIMGLDLPDGGHLTHGFMTDKKKISATSVFFESMPYK----------------
---------------VD-----------------------------------
PKTGLIDYERLRENAKIFHPRMIIAGISCYSRNLDYAKFREIADENNAYLLADMAHISGLVAAGAVPSPFEHCDIVTTTTHKTLRGV
RAGMIFFRKGVRSVG-KDGKPIMYNLESPINQAVFPGLQGGPHNHAIAGVAVALKQAAMPEFKTYIQQVIKNCQAMCKMMMDK-
GYHVVTDGSDNHLLLIDLRSKGINGS--KTEKILEEVSIACNKNTCPGDK-
SALNPSGLRFGTPALTSRGFVEKDFEKVTDFIDRAILLAVEIQTVSGK-----MLKDFKAKMVE-
EPFSSKIAALRAEVEAFAIAFPIPGLEDM--
>Bla_SHMT1_BLAG12000208_1
-------------------------------------------------------MAEPMQMTNGHA--------------------
-----------
HADWWDLTKPVNEVDPEITAIIRKEKDRQRRELEMIASENFASAACLQALGSCLNNKYSEGYPGQRYYGGTKFVDEIEVLCQKRALS
VYGLDPEKWGVNVQPYSGSPANFAVFTAVVEPHGRIMGLDLPDGGHLTHGFMTDKKKISATSVFFESMPYK----------------
---------------VD-----------------------------------
PKTGLIDYERMRENARIFHPKMLIAGISCYSRNLDYAKFREVADENNAYLLADMAHISGLVAAGVVPSPFEYCDIVTTTTHKTLRGV
RSGMIFFRKGVRSVG-KDGKPIMYNLETPINQAVFPGLQGGPHNHAIAGVAVALKQAAMPEFKTYQQQVIKNCQAMCKMLMDK-
GYHVVTDGSDNHLLLVDLRSKGINGS--KTEKILEDVSIACNKNTCPGDK-
SALNPSGLRFGTPALTSRGFVEKDFEKVTDFIDRAIQIAVEIQTVSGK-----MLKDFKAKMVE-
EPFSSKIAALRAEVEAFAIAFPIPGLEDM--
>Cin_SHMT1_XP_002127233.1 serine hydroxymethyltransferase, cytosolic [Ciona
intestinalis]
-------------------------------------------------------------MSCGDTKPTET---------------
------------
KESAWLEQPLEENDPEIYRIIRNEKERQRDGLELIASENFTSGAVLEALGSCLNNKYSEGYPGVRYYGGTENIDELERLCQKRALEV
FKLNPEEWGVNVQPYSGSPANFAVLTAIVEPHGRIMGLDLPDGGHLTHGFMTEKKKISATSIFFESMPYK-----------------
--------------VN-----------------------------------
PATGLIDYDQLEQNAKLFKPKVIIAGMSCYSRVIDYERIRKIADANKALVMADMAHVSGLVATGVIPSPFEHCQIVTSTTHKTLRGP
RAGIIFYRRGVKVPATDGKPAEMYNFEKPINEAVFPGLQGGPHNHAIAGVAVCLLQAKSPMFIEYQKNVVSNAQTLGKVLMDK-
GYDVVTGGTDTHLILVNLKSKGTDGN--RADKVLEAIGVACNKNTCPGDK-
AALRPSGLRLGSPALTSRGLNGKDFEKVADFIDRGVQLTVEIQNSLEPK---
ATFKDFRVKLYNDDVIIGKVKALKEEVTMFARTFPIPGLKYT--
>Hsa_SHMT2_NP_001159829.1 serine hydroxymethyltransferase, mitochondrial isoform 3
[Homo sapiens]
---------------------------------------------------------------------------------
MAIRAQHSNAAQTQTGEANRGWTGQESLSDSDPEMWELLQREKDRQCRGLELIASENFCSRAALEALGSCLNNKYSEGYPGKRYYGG
AEVVDEIELLCQRRALEAFDLDPAQWGVNVQPYSGSPANLAVYTALLQPHDRIMGLDLPDGGHLTHGYMSDVKRISATSIFFESMPY
K-------------------------------LN-----------------------------------
PKTGLIDYNQLALTARLFRPRLIIAGTSAYARLIDYARMREVCDEVKAHLLADMAHISGLVAAKVIPSPFKHADIVTTTTHKTLRGA
```

RSGLIFYRKGVKAVDPKTGREIPYTFEDRINFAVFPSLQGGPHNHAIAAVAVALKQACTPMFREYSLQVLKNARAMADALLER-
GYSLVSGGTDNHLVLVDLRPKGLDGA--RAERVLELVSITANKNTCPGDR-
SAITPGGLRLGAPALTSRQFREDDFRRVVDFIDEGVNIGLEVKSKT--A----
KLQDFKSFLLKDSETSQRLANLRQRVEQFARAFPMPGFDEH--
>Mmu_SHMT2_X2_NP_001239245.1 serine hydroxymethyltransferase, mitochondrial isoform 2 [Mus musculus]
--MVSFSLLRTTR---RCGQLV----
CMAARAQHSKVAQTQAGEAAGGWTGQESLSDSDPEMWELLQREKDRQCRGLELIASENFCSRAALEALGSCLNNKYSEGYPGKRYYG
GAEVVDEIELLCQRRALEAFDLDPAQWGVNVQPYSGSPANLAAYTALLQPHDRIMGLDLPDGGHLTHGYMSDVKRISATSIFFESMP
YK-------------------------------LN-----------------------------------
PQTGLIDYDQLALTARLFRPRLIIAGTSAYARLIDYARMREVCDEVRAHLLADMAHISGLVAAKVIPSPFKYADVVTTTTHKTLRGA
RSGLIFYRKGVRTVDPKTGKEIPYTFEDRINFAVFPSLQGGPHNHAIAAVAVALKQACTPMFREYSLQVLRNAQAMADALLKR-
GYSLVSGGTDTHLVLVDLRPKGLDGA--RAERVLELVSITANKNTCPGDR-
SAITPGGLRLGAPALTSRQFREDDFRRVVDFIDEGVNIGLEVKRKT--A----
KLQDFKSFLLKDPETSQRLANLRQQVEQFARGFPMPGFDER--
>Rno_SHMT2_NP_001008323.1 serine hydroxymethyltransferase, mitochondrial isoform 1 [Rattus norvegicus]
--MVPFSLLRTTRPLQRCGQLV----
CMAARAQHSEVAQKQAGEAAGGWTGQESLSDSDPEIWELLQREKDRQCRGLELIASENFCSRAALEALGSCLNNKYSEGYPGKRYYG
GAEVVDEIELLCQRRALEAFDLDPAQWGVNVQPYSGSPANLAAYTALLQPHDRIMGLDLPDGGHLTHGYMSDVKRISATSIFFESMP
YK-------------------------------LN-----------------------------------
PQTGLIDYDQLALTARLFRPRLIIAGTSAYARLIDYARMREVCDEVKAHLLADMAHISGLVAAKVIPSPFKYADIVTTTTHKTLRGA
RSGLIFYRKGVRTVDPKTGQEIPYTFEDRINFAVFPSLQGGPHNHAIAAVAVALKQACTPMFREYSLQVLRNAQAMADALLKR-
GYSLVSGGTDTHLVLVDLRPKGLDGA--RAERVLELVSITANKNTCPGDR-
SAITPGGLRLGAPALTSRQFREDDFRRVVDFIDEGVNIGLEVKRKT--A----
KLQDFKSFLLKDPETSQRLANLRQQVEQFARGFPMPGFDER--
>Xtr_SHMT2_NP_001007880.1 serine hydroxymethyltransferase, mitochondrial [Xenopus tropicalis]
--MLTFSLRRLAQPLRSC----------
CHVRSQHSQAWTQAGNQV--
WTGQESLAEGDPEMWDLVQKEKDRQCRGLEMIALENFCSRAALEALGSCLNNKYSEGYPGKRYYGGAEVVDKIELLCQQRALDAFDL
NPEKWGVNVQPYSGSPANFAAYTAVLQPHDRIMGLDLPDGGHLTHGYMSDVKRISATSIYFESMPYK--------------------
-----------LN-----------------------------------
PATGLIDYDQLEMTARLFRPKLIIAGTSAYARLIDYARMRKVCDEMKAYLLADMAHISGLVAAGVIPSPFEHADIVTSTTHKTLRGA
RSGLIFYRKGVKSVDKKTGKDVLYNLEDKVNFSVFPSIQGGPHNHAIAAVAVALKQASSPMFREYAVQVLKNAKSMAAALLSK-
GYTLVSGGTDNHLVLVDLRPKGIDGA--RAERVLELVSITANKNTCPGDK-
SALTPGGLRLGAPALTSRNFKEADFEKVVHFIDEGIRIGLDVKRKT--N----
KLQDFKNFLLEDHETVNRIADLRKQVEQFARSFPMPGFDER--
>Dre_SHMT2_NP_001116846.1 serine hydroxymethyltransferase, mitochondrial [Danio rerio]
--MLTLTLRQITRPL-------------
CRRVQRSSAAVCVRADGS--
WTGQESLSQDDPEMWDLLLKEKDRQCRGLELIASENFCSRAALEAQGSCLNNKYSEGYPGKRYYGGAEVVDQIELLCQKRALEAFDL
DPQLWGVNVQPYSGSPANFAAYTAVLNPHERIMGLDLPDGGHLTHGYMSDVRRISATSIYFESMPYK--------------------
-----------LN-----------------------------------
PKTGLIDYDQMELTAKLFRPKLIIAGTSAYARLIDYCRIKTLCSEINAYMLADMAHISGLVAAKAIPSPFQHADIVTTTTHKSLRGS
RAGLIFYRKGVRSVDKK-GKEIMYDLEEKVNFSVFPSLQGGPHNHAIAGVAVALKQATSPMFREYIAQVLKNSKAMAAALLDK-
GYTLVSGGTDNHLVLVDLRPQGMDGA--RAERVLELVSITANKNTCPGDK-
SALTPGGLRLGTPALTSRQLKECDFQKVVEFIHQGIQIGQDVKKKT--K----
KLSDFKSFLLEDAETVSRIADLRSRVEAFARPFPMPGFHDH--
>Bfl_SHMT2_XP_035657730.1 serine hydroxymethyltransferase, mitochondrial-like isoform X2 [Branchiostoma floridae]
--MLRSAATAVLTKAFRTNCQLLPRV----
TTSVSQQRWIMSATAQDQDEKVWTGQESLADSDPDMWGLLQKEKDRQLRGLELIASENFCSKAALEALGSCLNNKYSEGYPGQRYYG
GAEIVDQIELLCQQRAQQAFRLDPERWGVNVQPYSGSPANFAVYTALLNPHDRVMGLDLPDGGHLTHGFMTDTKRISATSIYFESMP
YR-------------------------------LN-----------------------------------
PQTGLIDYDKLEETARLFRPRMIIAGTSAYARLIDYKRMREICDEHGAYLLADMAHISGLVAAGVIPSPFEYADVVTTTTHKTLRGA
RAGLIFFRRGVKGQNKKTGKDIMYDFERRINFAVFPSLQGGPHNHAIAAVAVALKQAQTPMFREYQEQVMSNTKAMAESLMSK-
GYKLVSDGTDNHLVLVDLRPKGIDGA--RVERVCELASITCNKNTCPGDK-
SALTPGGLRLGAPALTSRCMKEDNFRQVVDFIDEAVQIGLQVKDKTG-P----
KMVDFKKFLLEDEETVGRISDLRARVESFARTFPMPGFDDH--
>Bla_SHMT2_BLAG12000293_1-BL15139_braLan3
--MLRSAATAVLTKALRTNGQLLPRV----
TTGVSQQRWVMSAAKQAQEEKVWTGQESLADSDPDMWGLLQKEKDRQLRGLELIASENFCSKAALEALGSCLNNKYSEGYPGQRYYG
GAEIVDQIELMCQQRAQQAFRLDPEKWGVNVQPYSGSPANFAVYTALLNPHDRIMGLDLPDGGHLTHGFMTDTKRISATSIYFESMP
YR-------------------------------LN-----------------------------------
AETGLIDYDKLEETARLFRPRMIIAGTSAYARLIDYKRMREICDEHGAYLLADMAHISGLVAAGTIPSPFEYADVVTTTTHKTLRGA
RAGLIFFRRGVKGQNKKTGKDIMYDFERKINFSVFPSLQGGPHNHAIAAVAVALKQAQTPMFREYQEQVMSNTKAMAEALMSK-
GYKLVSDGTDNHLVLVDLRPKGIDGA--RVERVCELASITCNKNTCPGDK-

```
SALKPGGLRLGAPALTSRCMKEDHFRQVVEFIDEAVQIGLQVQDKTG-P----
KMVDFKKFLLEDEETVGRISDLRARVESFARTFPMPGFDDH--
>Cin_SHMT2_XP_002126094.1 serine hydroxymethyltransferase, mitochondrial [Ciona intestinalis]
--------------------------------------------------------MLSRLYPRLIQQVYAVN------------
ASPLSR--FSS-----
KWTGRESLESEDPEILRIIKNEKDRQLRGLELIASENFCSRAAIEAMSSCLTNKYSEGYPGQRYYGGTENVDELELLCQKRALEAFH
LNPDEWGVNVQPYSGSPANFAAYTAVLKPHDRIMGLDLPDGGHLTHGFMTDAKRISSTSIYFESMPYR-------------------
------------LN-----------------------------------
PSTGYIDMDALENSAKLFRPRMIIAGASAYSRLIDYKRMREIADQHGAYVLSDMAHISGLVATQLVPSPFEHSDIVTTTTHKTLRGP
RAGIIFFRKGVRSVHKKTGKETMYDLESRINFAVFPSLQGGPHNPAIAAIAVALKQSMEPFFKEYQIQTLKNAATMASELTAR-
GYNLVSGGTDNHLVLVDLRPKGIDGA--RTEKVLELASVTVNKNSVPGDK-
SALMPGGLRLGAPALTSRDFVEEDFVKVVDFLDKGVEIAIEAKKKT--K----
KLADFKSFIETNPETVEKISNLRNEVEKFARSFPMPGFEDH--
>Pfl_SHMT2_XP_070547159.1 serine hydroxymethyltransferase, mitochondrial-like [Ptychodera flava]
------------------------------MRGSVMATQGHWLSHSTLRFTALSFNMAVRRFSPAVSRIISRRF---------
QSTQS--
AAAERQAETQAAWNGQETLAESDPEIMALVWEEKDRQKRGLELIASENFASRAVLESLGSCLNNKYSEGYAGQRYYGGNEIIDKVET
LCIQRALEAFDLDPAKWGVNVQPYSGSPANFAVYTALLNPHDRIMGLDLAHGGHLTHGFMSDTKRVSATSIFFESMPYR--------
-----------------------LN-----------------------------------
PSTGYIDYDKLQETARLFRPRLIIAGTTAYSRLLDYPRFREICDESNAVMMADMSHISGLVAGKVIPSPFDYADVVTSTTHKTLRGP
RAGMIFFRKGVKGVNKKTGKEIKYDFESKVNGAVFPALQGGPHEHAIAGIAVALKQAMSPQFREYQAQVLKNAQTMAECLKNKYDWD
LVSGGTDNHLVLANLRPKGIDGS--RAERVLEMSSITVNKNTTPGDK-
SALNPGGLRIGAPALTSRNFKQKEFEKVSDFLNQGIEITREAKQKTGTA----
KLKEFKEFIMQDDVTQKKISELREEVETFARQYPMPGFEDH--
>Pfl_SHMT1_XP_070580019.1 serine hydroxymethyltransferase, cytosolic-like [Ptychodera flava]
--------------------------------------------------------MEDTSMDKPSYDKING---------------
-----------
HDAEWTLQDPLKEDDPEMYELLIKERNRQKRGLELIASENFASKAVLQTLGSCLNNKYSEGYPGVRYYGGNEVIDDIERLCQKRALE
VYDLDPEKWGVNVQPYSGSPANFAVYTGVVEPHGRIMGLDLPDGGHLTHGFFTEKKKISATSIFFESMPYK----------------
---------------VN-----------------------------------
PESGLIDYEKLQDNAKLFKPKLIIAGVSCYSRHIDYATMRTIADENGSLLLADMAHISGLVAAKVIPSPFDHCDIVTTTTHKTLRGP
RSGMIFYRKGVRKTL-NNGKQVMYDLEEKINGAVFPGLQGGPHNHAIGAVAVALQQAKRPAFRTYCEQVISNAKTMSDELISR-
GYTIATGGTDNHLILLDLRPIGLDGN--RAEKVLEAVSIACNKNTCPGDK-
SALKPGGLRLGAPALTSRKFINNHFVKVVEYIHRGIQLTQEIKAASGP-----
TLKEFKNTLYNDPNFGDKIKVLREEVEEFAAQFPLPGETLP--
>Dme_SHMT_XA_NP_572278.1 serine hydroxymethyl transferase, isoform A [Drosophila melanogaster]
MQRARSTLTQKLRFCLSRDLNTKVGNPVNFETGKLSGALTRIAAKKQPSPTPFLPAIRRYSDSKQSTLKN-----------------
-----------
MADQKLLQTPLAQGDPELAELIKKEKERQREGLEMIASENFTSVAVLESLSSCLTNKYSEGYPGKRYYGGNEYIDRIELLAQQRGRE
LFNLDDEKWGVNVQPYSGSPANLAVYTGVCRPHDRIMGLDLPDGGHLTHGFFTPTKKISATSIFFESMPYK----------------
---------------VN-----------------------------------
PETGIIDYDKLAEAAKNFRPQIIIAGISCYSRLLDYARFRQICDDVGAYLMADMAHVAGIVAAGLIPSPFEWADIVTTTTHKTLRGP
RAGVIFFRKGVRSTK-ANGDKVLYDLEERINQAVFPSLQGGPHNNAVAGIATAFKQAKSPEFKAYQTQVLKNAKALCDGLISR-
GYQVATGGTDVHLVLVDVRKAGLTGA--KAEYILEEVGIACNKNTVPGDK-
SAMNPSGIRLGTPALTTRGLAEQDIEQVVAFIDAALKVGVQAAKLAGS----
PKITDYHKTLAENVELKAQVDEIRKNVAQFSRKFPLPGLETL--
>Mgi_SHMT_XP_011435353.3 serine hydroxymethyltransferase [Magallana gigas]
--------------------------------------------------------MADVKMNGGGD--------------------
--------------
YTNLQADIAVDDPEMYNLIKKEKDRQMRGLELIASENFTSKAVLQCLGSCLTNKYSEGQPGQRYYGGNEYIDQVERLCQKRALDLYG
LSPEEWGVNVQPLSGSPANFAVYTGVVGPHGRIMGLHLPDGGHLSHGFMTPTKKISATSVYFESFPYR-------------------
------------VS-----------------------------------
PKTGLIDYDQLHENAKLFLPNMIIAGVSCYSRHLDYKRFREIADENGAYVLADMAHVSGLVATGVAPSPFEYCDIVTTTTHKTLRGP
RSGMIFFRRGVRKLK--NGKEEKYDLERRINEAVFPGLQGGPHNHQIAGVAVALQQAARPEFKTYQQQVVKNAQAMCKAFMEK-
GYTVVTGGTENHLILIDLRPNGMDGA--RAERVLECVSIALNKNTCPGDK-
SALKPSGLRIGAPALTSRDFKEKDFEQVVEFIHKGLVITKEAMQDCGP-----
LLKDFKAKLDADQTIKAKIDNLRSDVENFALKFPMPGIDNW--
>Mgi_SHMT2_XP_011420488.3 serine hydroxymethyltransferase, mitochondrial isoform X1 [Magallana gigas]
-------------------------------------------------------------MARVGILSFSIRD----------
MLSRRLLTR---
QAIRTLWTGRESLEEDDPELMSIIKQEKKRQVNGLELIASENFASRSVQEALGSCLTNKYSEGYPGARYYGGNVFIDKVESLCQTRA
LEAFRLDPHQWGVNVQPYSGSPANFEAFTALLKPHDRIMGLDLPDGGHLTHGFMTDTRRISATSVFFESMPYR--------------
-----------------ID-----------------------------------
PKTGYIDYDKLRESARLFRPKLIIAGTTAYSRLLDYKAYREICDEVNAYMLADMAHISGLVAADVIPGPFEHADVVTSTTHKTLRGP
```

RSGMIFYRKGKKGVDKK-GNDVMYDLEKKINNAVFPALQGGPHQHQIGALAVALKQAKSPEFKEYQLQVIKNAKVMAKVLLDK-
GYSVVSGGTDNHLVLVDLKSKGTDGA--RVERILELCEISVNKNTCAGDK-
SPMTPGGLRIGAPAMTSRGMKEKDFEKICEFLDRGVQIGINAKKYS--K----
TLKEFRHAVIENEDIQGQINKLRGEVESFASQFPMPGLEER--
>Obi_SHMT2_XP_052830111.1 serine hydroxymethyltransferase, mitochondrial isoform X1 [Octopus bimaculoides]
--MVMCREVKVWDETSALLQ----------
LLPPSAATITKVGQHRTIWTGQEELKDVDPEMHDLLQKEKSRQIYGLELIASENFASRAVIDVTGSCLTNKYSEGYPGARYYGGNIY
IDQIERLCQKRALECFRADPERWGVNVQPYSGSPANMAVYTAVLNPHDRIMGQDLPDGGHLTHGFMTDTKRISATSIFFESMPYK--
-----------------------------IN-----------------------------------
PSTGLVDYDKLHEHAKLFRPRLIIAGTSAYSRLLDYKRFREICDDVNALLLADMAHISGLVAAAVIPSPFESADLISTTTHKSLRGP
RSGMIFYRKGVKSVNKKTGEIKMYDLEAKVNNAVFPALQGGPHQNQIGALAVALKQAMDPEFKDYQVQVMKNAKTLAKCLNAK-
GYKLVSDGTDNHLVLVDMKSKGIDGA--RAERILELSGITTNKNTCPGDK-
SAMVPGGIRLGTPALTSRGFKEEDFELVVDFFDQAIQLGQEVKKKT--S----ELAMFYVCA-------------------------
----
>Obi_SHMT_XP_052834316.1 serine hydroxymethyltransferase [Octopus bimaculoides]
---MSTTNGYE--------------------

GWCLQDDISQNDPQMYEFIKQEKLRQKQGLEMIASENFTSKAVMQALGSCLTNKYSEGYPGQRYYGGNEIIDQVESLCQKRALEVFK
LNPEEWGVNVQPLSGCPANFAVYTGVVGPHGRIMGLHLPDGGHLSHGFMTNKRKVSATSVFFESFPYK-------------------
------------VN-----------------------------------
PVSGLIDYDALETNAHLFNPMLIVAGVSCYSRHLDYARFRKISNAVDCLLMADMAHISGLVAAGVVPSPFEHCDIVTTTTHKSLRGP
RSGMIFYRK---------------------------------------ALAVALKQAKTEEFVQYQKQVLCNAHVMAEEFIAR-
GYTLATGGTENHLVLLDLRPDQLDGA--SVERILEEINIAVNKNTCPGDK-
SALKPSGIRIGTPALTSRMFKENDFKQVVEFIHQGILLCKEIYSKCGS-----
SFSEFKAFLEKDADIQKKVANVREQVKQFAEKFPMPGLDDM--
>Dma_SHMT_XP_032782924.1 serine hydroxymethyltransferase [Daphnia magna]
---MSEPNTKEE-----------------
-----------MVKK--
LNETLEVADPEIFELITKEKHRQSKGLEMIASENFTSKAVLQALSSCLHNKYSEGLPGMRYYGGNEFIDQIEVLCQKRCLEAYGLNA
EQWGVNVQPYSGSPANFAVYTGIVEPHGRIMGLDLPDGGHLTHGFFTATKKISATSIFFESMPYK----------------------
---------SD-----------------------------------
PKTGLIDYDQLAITARLFKPKLIIAGISCYSRNLDYAKFRAIADDVGAYLMADMAHVSGLVAAGVAPSPFPFCDIVTTTTHKTLRGP
RAGVIFFRRGPTNVP-GSS----YDFENRINQAVFPGLQGGPHNHAIAAIAIAMKHAKTEDFKSYQQQVVKNAQELCRGLQDL-
GYKIVTDGTDNHLILMDLRTVGLTGS--KGEKILEEIGIACNKNTVPGDK-
SAFNPSGIRLGTPALTTRGLLEADIARVVQFINKGLQLALEISAISG-----
PKLVDFKRVLLENPQINAKVLQLRSEIESFALDFPMPGYDI---
>Pte_SHMT_XP_042908860.1 serine hydroxymethyltransferase [Parasteatoda tepidariorum]

MDPILNQTLDESDPDLLKLLKKEKERQSRGLEMIASENFTSRSVLQCLSSCLHNKYSEGYPGQRYYGGNEFIDEVERLCQQRALQTY
GLNPEEWGVNVQPYSGSPANFAVYTAVVEPHGRIMGLDLPDGGHLTHGYFTEKKKISATSMFFESMPYK------------------
-------------LD-----------------------------------
PSTGLINYDQLEELVKLFKPKLIIAGISCYPRDLNYKRFREIADINNAYLMGDMAHVSGLVAAKLAPSPFEYCDIVTSTTHKTLRGP
RAGLIFYRKGVRKVT-KAGEEK-YDLEDKINQAVFPGLQGGPHNNSISGIATALKQAQTKAFYEYQSQVLANSKCLAKGLQSR-
GYTVVTGGTDNHIVWVDLRPIGLNGS--RAEKVLEDISIACNKNTVPGDK-
SALNPGGIRLGTPALSTRGLKESDMDKVAEFLHKGFQLALEVKSKSG-----PLLKDFKSTLE-
NSEFQEKVKNLKDAVEGFASRFPLPGYEGY--
>Cel_SHMT_NP_001379624.1 Serine hydroxymethyltransferase [Caenorhabditis elegans]
--MADRQVHTPLAKVQRHKY-------------

TNNENILVDHVEKVDPEVFDIMKNEKKRQRRGLELIASENFTSKAVMDALGSAMCNKYSEGYPGARYYGGNEFIDQMELLCQKRALE
VFGLDPAKWGVNVQPLSGSPANFAVYTAIVGSNGRIMGLDLPDGGHLTHGFFTPARKVSATSEFFQSLPYK----------------
---------------VD-----------------------------------
PTTGLIDYDKLEQNAMLFRPKAIIAGVSCYARHLDYERFRKIATKAGAYLMSDMAHISGLVAAGLIPSPFEYSDVVTTTTHKSLRGP
RGALIFYRKGVRSTN-AKGVDTLYDLEEKINSAVFPGLQGGPHNHTIAGIAVALRQCLSEDFVQYGEQVLKNAKTLAERMKKH-
GYALATGGTDNHLLLVDLRPIGVEGA--RAEHVLDLAHIACNKNTCPGDV-
SALRPGGIRLGTPALTSRGFQEQDFEKVGDFIHEGVQIAKKYNAEAG-----
KTLKDFKSFTETNEPFKKDVADLAKRVEEFSTKFEIPGNETF--
>Tad_XP_002109673.1 uncharacterized protein TRIADDRAFT_53932 [Trichoplax adhaerens]
---MASFLKTTRSLGN---------
LPAQVRSILFARGLPVRTAWTGLQSISEDDPELFDIIRREKSRQRGDLELIASENFTSRAVMNALGSCLTNKYSEGYPGQRYYGGNQ
CIDEIELMCQRRALEAYDLDPEKWGVNVQPYSGSPGNFAVYTGLLKPHSRVMGLDLPDGGHLTHGFMSGKVRISATSIYFESLAYHS
HMHNSPLGRSETKVALRLPDEGRCNRRIYALDNHHSVIAKNGNPACRRYLHRRRNNIDMIGIRMYAKPDTGEVDYDALQKQAKAFVP
EMIIAGTSAYSRLLDYQKFREICNDVKAILMADMAHISGLVAAKVVPSPFEYADVVTTTTHKTLRGP--------------------
-------SKINKAVFPGLQGGPHNNVIAGVAIALRQAKTPEFVEYQKQVLKNCKAMANALLNK-
GYTLISGGTDNHLILVDLRPKGVDGS--RTERVLELVNISTNKNTCPGDK-
SALFPGGMRLGTPALTSRDFKEKDFEQVVEFIERGVQITYEAKQKTG------
TLKEFKEFVISDPDITAKISALRQEVKEFAEQFPMPGQDY---

```
>Tad_XP_002109683.1 uncharacterized protein TRIADDRAFT_20682 [Trichoplax adhaerens]
---------------------------------------------------------------------------------------
-------
MADNYQFDSSLKNSLQEEDPEIYHLICKEKKRQRLGLELIASENYASRATLQALGSCLNNKYSEGYPGARYYSGTQVVDDIELLCQR
RALELFGLDREQWGVNVQPYSGSPANFAVYTALLQPHDRIMGLDLPDGGHLTHGYMNDTKRISASSIYFESMPYK------------
----------------IN--------------------------------------
PTTGLIDYDQLEANAKLFRPKLIIAGISSYCRHLDYARIRQIADQQKAYVLSDMAHVSGLVAAKLAPTPFQYSDVVTTTTHKTLRGP
RSALIFYRKGIRHHDQS-GQPIYYDLQDKINFAVFPALQGGPHNHAIAAVAVALKEAQSDKFIQYQKQVLSNCQTLSDGLIAL-
GYTLVTGGSDNHLILLDLRPQKLNGA--RAVEVFERVHISANKNTCPGDK-
NALIPSGIRFGTPALTSRGLSCQDMVKIVQFIHRALQIAIDATSTVAGK----SIKDYKATLDQE-
EYQAKIQQLAEEVLEFSSQFPTPGSDVI--
>Pmi_SHMT1_XP_038064736.1 serine hydroxymethyltransferase, cytosolic-like [Patiria
miniata]
------------------------------------------MNGTAVETSVEPTVAKRQKLSNGGEVHLDIMPANDTT--------
-------
VVTPAPEGWFGSEPLATNDPAIANIISKEKDRQRRGLELIASENFTSRAVLETLGSCLQNKYCEGYPGNRYYGGTEHYDELELLCIK
RAQEAYGLNPDEWGVNVQPYSGSPANFAVFTAVIGPHGRIMGLDLPDGGHLTHGFMTSRKKISATSVYFESMPYK------------
-------------------VN-----------------------------------
PETGYIDMDRLEENARLFHPQVVIAGISCYSRNLDYARFRKIADQHDAYLMADMAHISGLVASGAVPSPFDHCDIVTSTTHKTLRGP
RAGIIFFRKGVRKTL-KNGTQVMYNLEKTINEAVFPGLQGGPHMHAIAGVAVALKQTMKPEFKTYAANVIQNAQVMCKSLVER-
GYKVVTGGTDNHLLLVDLRPIGLDGA--RCEKILEDISVVCNKNTCPGDQ-
SALKPSGLRFGTAALTSRTLTAADFEKVVDFIDRGIKLTKEVAAISGP-----
TLKEFRETVATNEEVKKKIAAMREEVETFAMKFPLPGF-----
>Pmi_SHMT2_XP_038044129.1 serine hydroxymethyltransferase, mitochondrial-like [Patiria
miniata]
--------------------------------------------------------MASRSLSRLLSLRPAIQN---------
VSRATYGQQATLQSQQVPDWTGQEILSEVDPEILSLIREEKQRQVRGLELIASENFCPQASLNVLSSCLHNKYSEGLPGNRYYGGTE
VIDKVERLCQKRALEAFDLDPEKWGVNVQPYSGSPANLAVYTGLLAPHDRIMGLDLPHGGHLTHGFMTDTTRISATSIFFESMPYR-
------------------------------LD-----------------------------------
IKTGLVDYDKLEETSRLFRPRLIISGYSAYPRRLDYKRFREICDGVKAIMMSDMAHISGLVAAKILPSPFDHSDIVTTTTHKTLRGP
RSGLIFFRRGKKGVSKKTGKDIMYDYEKRINHAVFPALQGGPHNNNIGGVAVALKQTMTPAFKEYQRQTVLNAKTMAQALLAK-
GYSLVSGGTENHLVLVDLHSKGVDGA--QAEHILELCAISVNKNTCPGDK-
SALHPSGLRLGSPALTSRNFKEGDFLQVIDFIDRSIAIGQEAKKISG-A----
NNKVYYEVVETNKEFLKKIAKLRLEVEDFAAQFPMPGYDNF--
>Ame_XP_071965043.1 serine hydroxymethyltransferase, cytosolic-like [Antedon
mediterranea]
-------------------------------------------------------------MANGD---------------------
------------
SKAWFGDEPLEQNDPEIYNIILKEKDRQRRGLELIASENFASKAVLEALGSCLNNKYSEGYPGTRYYGGTKFIDELETLCQNRAREA
FGLTAEKWGVNVQPYSGSPANFAVYTALVEPHGRIMGLDLPDGGHLSHGFMTDKRKVSATSIFFESMPYK-----------------
--------------VD-----------------------------------
PQTGLIDYEQLQVNAKLFHPKLIIAGISCYSRNLDYAKLREIADENNALLLADMAHVSGLVAASLVANPFDYCDIVTSTTHKTLRGP
RAGIIFFRRGVRRTL-KNGNEEMYNLEKPINEAVFPGLQGGPHNHAIAGVAVALKQTMDPSFKAYGKEVIQNARVMAKCLMDR-
GYHIVTGGTDTHLLLLDLRSKGLDGN--RADKVLEEISIACNKNTCPGDK-
SALRPSGLRFGTPALTSRNLKAADFEKVVDFIDKGISIALEIKAASGP-----LLKEFIATMES-
ETYQKKIATLRDEVETFAVKFPLPGKDVL--
>Ame_XP_071941370.1 serine hydroxymethyltransferase, mitochondrial-like [Antedon
mediterranea]
-----------------------------------------------------------MSFPSLRLLR--------------
TSSCRGILNSALRSSSSDTWTLQEPLTEDDPEISALIKEEKKRQVRGLELIASENFCSRAAIEAMGSCLTNKYSEGYPGQRYYGGTY
VIDKIELLCQQRALDAFDLDPEKWGVNVQPYSGSPANFAVYTGLLKPHERIMGLDLPHGGHLTHGFMTDTKRISATSIYFESMPYR-
------------------------------LD-----------------------------------
ETTGLIDYDKLAETANLFRPKLIIAGYSAYPRLLDYARFRKICDDQKAILLSDMAHISGLVAAKVLPTPFEHSDVVTTTTHKSLRGT
RAGMIFYRRGVKGISKKTGKELKYDFEDKINFAVFPSLQGGPHNPNIAGVAVALKQASSEKFVDYQKQVLKNANAMAEELIAL-
GYTLVSGGTENHLALLDLRPKGVDGA--RAEKVLELASITVNKNTCPGDK-
SALVPGGLRLGAPALTSRNFKDNDFREVAKFLDRGCQITIDAKAQAG-K----
TLAEFIKFVQTDEKIKSRISELRKEVEEYASTFPMPGFDDH--
>Hpa_XP_064397821.1 serine hydroxymethyltransferase, mitochondrial-like isoform X1
[Halichondria panicea]
---------------------------------------------------------MQRLFPSALCLVRTTE--------------
LLLQR--
SSVRMASQWTGQEPLAQTDPEIKSIIQNEKQRQKLGLELIASENFCSRAVQECLGSCLTNKYSEGYPGQRYYGGNECIDEIERLCQK
RALDAYGLSPDQWGVNVQPYSGSPANFAVYTGLLAPHDRIMGLDLPDGGHLTHGFMTGKKKVSATSKYFESMAYH------------
-------------------VN-----------------------------------
PQTGLIDFEGLRKSAKDFLPRMIIAGTSAYSRVLDYARFRDICNEVGAYLMSDMAHISGLVATGVHPSPFEFSDVVTTTTHKTLRGP
RSGIIFYRRGVKGQDKA-GKDIMYDYERKINSAVFPGLQGGPHNHQIAAVAVALKVASTPEFKEYQLQVVSNARTLAKALADR-
GYNIVTGGTDVHLFVVDLRSKSVDGS--
LAEHILELISISVNKNTVPGDTGSALHPSGLRIGTPALTSRYMKDSDMGNIAGFIDEGIKIVAEVKGQLSKP--
NLTVKQFNAHLAKDEATQKKVSELRGKVEVFATQFPMPGFEDH--
```

```
>Aqu_XP_003387864.2 PREDICTED: serine hydroxymethyltransferase, cytosolic-like isoform
X1 [Amphimedon queenslandica]
------------------------------------
MSCKLFWIMSITCMGATIVGVFLKKAESLRRTRERCITVITKAAKVTFRALDRRFQQVMASSSWTLQEPLEEDDPEIFELIKKEKQR
QRNGLELIASENFASRSVLEAMGSCLNNKYSEGYPGQRYYSGNEVIDKIESLCQKRALEAFGLDPKEWGVNVQPYSGSPANFAAYTG
ILNPHDRIMGLHLPDGGHLTHGFMRGSQRVSATSLYFESMPYH-------------------------ID----------
----------------------
PKTGIINYDQLEMFAKSFHPRMIIAGTSAYSRLIDYQRIRKICDDNGAYLLSDMAHISGLVAARVIPSPFEYSHVVTTTTHKTLRGA
RSGMIFYRRGVKEINKQ-GQEVMYDFEKKINAAVFPALQGGPHNHAIAGVAVALKQACRPEFRVYQEQVVKNAKVLAESLMGF-
GYHIVSDGTDTHLMLLDLRGTGMDGAGGKADRVLELASVTANKNTVPGDR-
NAMNPSGLRLGTPALTSRFMKEDDMKQVGAFIHEGVQIACEVNQKLEAAGTKPTNKVFKEFVVSDAPTIAKIEELRGRVEEFAKKFP
IPGFDEH--
>Sko_XP_002740742.1 PREDICTED: serine hydroxymethyltransferase, mitochondrial-like
[Saccoglossus kowalevskii]
---------------------------------------------------MAARRLTPVASRIVFRRF--------
RSSKS--
FTASGESIEPVIWTGKETLAESDPEMMALISEEKDRQVRGLELIASENFASRAVLESVGSCLNNKYAEGYPGQRYYGGNETIDKVER
LCQSRALEAFDLDPEKWGVNVQPYSGSPANFAVYAGLLNPHDRIMGLDLAHGGHLTHGFMSDTKRVSATSIFFESMPYR-------
---------------------LN--------------------------------
QQTGYIDYDKLEMTAKLFRPRLIIAGTTAYSRLLDYPRFRQICNDTNSVMMADMAHISGLVAAKVIPSPFEYADVVTSTTHKTLRGP
RAGVIFFRRGVKGVDKKTGKEIKYDYESRINGAIFPALQGGPHEHAIGGVAVALKQAMSPQFREYQTQVLKNAKALADSLMKLYGWD
LVSGGTDNHLVLANVRPLGVDGA--RAERVLELCSITVNKNTTPGDK-
SALNPGGLRLGAPALTSRGFKESDFREVAGFLDRGVKITHDAKQKTG------
KLKEFREFIMKDDAILQKMQGLRTDVEDFARQFPMPGFDER--
>Sko_NP_001171839.1 serine hydroxymethyltransferase, mitochondrial-like [Saccoglossus
kowalevskii]
------------------------------------------------------MYVDNPNYDKMNG--------------
----------
EKEEWSLNDPLKTNDPEMYELLIKERNRQKRGLELIASENFASKAVLQALGSCLNNKYSEGYPGLRYYGGNEYIDDIERLCQKRALD
LYDLDPSKWGVNVQPYSGSPANFAVYTAIVEPHGRIMGLDLPDGGHLTHGFFTEKKKISATSIFFESMPYK---------------
--------------VN--------------------------------
AETGLIDYDRLRENAGLFKPRMIIAGVSCYSRNLDYATLREICDENGSYLMSDMAHISGLVAAKVVPSPFDYSDVVTTTTHKTLRGP
RSGMIFYRKGVRKVL-KDGTEVKYDLEEKINAAVFPGLQGGPHNHAIGGVAVALKQAQSPSFRTYQEQVVSNAKTMAASLIKK-
GYTIVTGGTDNHLILLDLRSVGLDGN--RAEKVLEAISVACNKNTCPGDK-
SALRPGGVRLGAPALTSRKFKNQDFEKVCEFIDRGLQLALEIKAVSGP-----
LLKDFKNLLYKDAKFQEKVSAIREEVEAFAVNFPLPGETLPED
>Pca_XP_025080556.1 serine hydroxymethyltransferase, mitochondrial-like [Pomacea
canaliculata]
--------------------------------------------------------MYFSSRLVFAPRQ---------
KLQNVAVT----
LWARHGSTGREPLAESDPEAVEIIRKEKFRQTHGLELIASENFASRAVLEALGSCLNNKYSEGYPGARYYGGNVFIDEMERLCQKRA
LEAFKLDPEKWGVNVQPYSGSPANFAVFTAVLQPHDRLMGLDLPDGGHLTHGFMSDTKRVSATSLFFESMPYK-------------
----------------LD--------------------------------
PTTGYIDYEKLRETARLFRPRLIIAGTTAYSRLLDYEYFRKVCNEVNAVMLADMSHISGLVAAGVIPSPFEYADIVSSTTHKTLRGP
RSGIIFFRKGIKEIDKKTGKEIMYDFEKKINNAVFPALQGGPHDHQVAGVAVALLEAMQPEYKEYQKQVLKNAKAMCSALLAR-
GYKVVSGGTENHLVLVDLRPHGTDGA--RVERVLELCYITVNKNTCAGDK-
SAMTPGGLRLGAPALTSRGLKEADFERVVELLDKGVKIALNVQKQT--K----
SLKEFKAYVLENHDALMQMESLRSEVQSFAGKFNMPGFDDR--
>Pca_XP_025091629.1 serine hydroxymethyltransferase, cytosolic-like [Pomacea
canaliculata]
-------------------------------------------------------MTEDCR-------------------
--------------
FWLQDDIRDDDPEIYELIRAEKERQRRGLELIASENFASKSVLQALGSCLNNKYSEGQPGQRYYGGNEIIDKVELLCKKRALDAYNL
NNEEWGVNVQPLSGSPANFAVYTALVEPHGRIMGLHLPDGGHLSHGFMTNQKKVSATSLFFESFPYK------------------
----------LD--------------------------------
PATGLIDYDKLAENAKLFLPKMIVAGVSCYSRNLDYKRFREIADEVGAYLLADMAHVSGLVAAGVVPSPFEYCDVVTTTTHKSLRGP
RSGMIFYRKGVRKET-KNGEKVMFDLERKINEAVFPGLQGGPHNHQIAAVAVSLKQAQTPEFKLYQQQIVSNAKAMAKALQNK-
GYHIVSGGTDTHLVLVDLRPQKLDGA--RGEKVLEEIGLAVNKNTCPGDK-
SALKPSGLRLGTPALTSRNFKEADFEKVIDFFHEAIELTKEINSSCGP-----
LLKEFKAKVDEDPVIRSKIKDLKQRVENFAVSFPMPGFNDW--
>Cte_ELU01860.1 hypothetical protein CAPTEDRAFT_151243 [Capitella teleta]
---------------------------------------------------MFPAAIRQIALRPVLRQR-----------
------
PLSVSASQWTGQDSLVDDDPKVADIISREKKRQMRGLELIASENFASRAVLEALGSCLSNKYSEGYPGARYYGGTECVDELELLCQK
RALDLYGLDPEEWGVNVQPYSGSPANFAVYTALLQPHDRVMGLDLPDGGHLTHGFMTDTKRISGTSIYFESMPYR-----------
-----------------LN--------------------------------
PSTGLIDYDKLRENAALFRPKMIIAGTSAYSRLLDYKAFREICDQHNAYLLADMAHISGLVAAKVIPGPFEYADVVTTTTHKTLRGP
RSGMIFYRKGVKGVDKK-GKEIKYDLQKRIDFAVFPSLQGGPHQHQIAGIAVALRQATSPEFVAYQKQVLANCKVMANTLMAK-
GYSLIAGGTDNHLVLLDLRPKKIDGA--RAERVCELCSITVNKNTCPGDK-
```

SALVPGGLRLGAPALTTRGMKEKDFEAVVGFIDEAVQIAQGVKAQTG------
NLKEFKAFLLADAGTQSKIADLKSRVEAFADGYIMPGFQDR--
>Cte_ELU03449.1 hypothetical protein CAPTEDRAFT_180856 [Capitella teleta]
---MTTNGNQ------------------

KWDLQDDISVVDPAMAEILNKEKERQVCGLEMIASENFASRAVLQALGSCLNNKYSEGQVGQRYYGGNEFIDEMETLTKNRALEVYG
LSPEEWGVNVQPLSGSPANFAVYTALVEPHGRIMGLDLPDGGHLSHGFFTATKKISATSIFFESLPYR------------
-----------LN--------------------------------
PETGLIDYDKLAENARLFKPRMIIAGMSCYSRNLDYKRFREISDENNSYLLADMAHISGLVAAGVVPSPFEYCDVVTTTTHKTLRGP
RSGMIFYRKGVRKVT-AKGDKVMYDLEKKINEAVFPGLQGGPHNHAIAGVGVALGLALRPDYKVYQQQVVTNCQTMVKQLMKL-
GYLVVSGGTDNHLALVDLRPMNTCGA--RAEKVLEDISIAVNKNTCPGDK-
SALRPSGLRLGTPALTSRNMKEPEILKVVDFIHRAITLTLEIQANCGP-----
TVREFKAKLAEDADIQKKVKALRDEVETFAKAFPMPGHIGAYA
>Sca_XP_039253911.1 serine hydroxymethyltransferase, mitochondrial-like [Styela clava]
---MFSSKLSCLQRSLKLGNN------
AKVIWTRPLSTSVFSLSQTKWNWTGNETLEQDDPEINSLIKQEKDRQVRGLELIASENFASRAALEAMGSCLTNKYSEGYPGQRYYG
GTEIVDKIELLCQKRALEVFRLDPEKWGVNVQPYSGSPANFAAYTAVLNPHDRIMGLDLPDGGHLTHGFMTDTKRISATSIYFESMP
YR-----------------------------LN--------------------------------
PNTGTIDYDSLEANAKLFRPRLIIAGASAYSRKIDYERMKKITNQHNAYLLSDMAHISGLVAADLMPSPFEHSDIVTTTTHKTLRGA
RSGLIFYRKGVRSTSKKTGKEIKYDLEDRINFAVFPSLQGGPHNYAIAGVAVALRQAMSPEFKEYQIQTMKNAKAMSDALQSL-
GYNVVSGGTDNHLVLVDLRPKGIDGA--RTEKVLEAASITANKNSVPGDK-
SALIPGGLRLGAPALTSRHFVEDDFRKVVDFLNRGVEIAVEAKTRT--K----
KLADFINFIKSDAEVQRKISELRKEVETYARKFPMPGFPDH--
>Sca_XP_039260153.1 serine hydroxymethyltransferase, cytosolic-like [Styela clava]
--MSG--NKQIAG-------------

GDGEWLQQSLEDDDQEIYQIIRNEKERQRCGLELIASENFASAAVLQALGSCLNNKYSEGYPGVRYYGGAEYIDEMERLCQRRALEL
FKLNKDEWAVNVQPYSGSPANFAVYTAIVQPHGRIMGLDLPDGGHLTHGFFTEKKKISATSIFFESMPYK----------
-------------VN--------------------------------
QETGLIDYDKLAENAKLFKPKIIIAGVSCYSRHLDYKRMREIADENKAYLMADMAHISGLVAAGIVPSPFEHCHIVTTTTHKTLRGP
RSGMIFCRKGVKSVDPKGN-KEMYNLEKPINEAVFPGLQGGPHNHAIAGVAVALKQAMTDKFIQDQKQTIANAQHMAKLLVDR-
GYNVVTGGTDNHLMLVNLKSKGTDGN--RADKVLEAIGVACNKNSCPGDT-
SALRPSGLRLGSPALTSRGFKEADFDKVADFIDRGIQLTLEIQQVIGPK---
APFKDFKDKLYKDEDICAKVKSLKDEVASFASAFPIPGFPLL--
